# Effect of analytical treatment interruption and reinitiation of antiretroviral therapy on HIV reservoirs and immunologic parameters in infected individuals

**DOI:** 10.1371/journal.ppat.1006792

**Published:** 2018-01-11

**Authors:** Katherine E. Clarridge, Jana Blazkova, Kevin Einkauf, Mary Petrone, Eric W. Refsland, J. Shawn Justement, Victoria Shi, Erin D. Huiting, Catherine A. Seamon, Guinevere Q. Lee, Xu G. Yu, Susan Moir, Michael C. Sneller, Mathias Lichterfeld, Tae-Wook Chun

**Affiliations:** 1 Laboratory of Immunoregulation, National Institute of Allergy and Infectious Diseases, National Institutes of Health (NIH), Bethesda, Maryland, United States of America; 2 Ragon Institute of MGH, MIT, and Harvard, Cambridge, Massachusetts, United States of America; 3 Critical Care Medicine Department, Clinical Center, NIH, Bethesda, Maryland, United States of America; 4 Infectious Disease Division, Brigham and Women’s Hospital, Boston, Massachusetts, United States of America; University of North Carolina at Chapel Hill, UNITED STATES

## Abstract

Therapeutic strategies aimed at achieving antiretroviral therapy (ART)-free HIV remission in infected individuals are under active investigation. Considering the vast majority of HIV-infected individuals experience plasma viral rebound upon cessation of therapy, clinical trials evaluating the efficacy of curative strategies would likely require inclusion of ART interruption. However, it is unclear what impact short-term analytical treatment interruption (ATI) and subsequent reinitiation of ART have on immunologic and virologic parameters of HIV-infected individuals. Here, we show a significant increase of HIV burden in the CD4^+^ T cells of infected individuals during ATI that was correlated with the level of plasma viral rebound. However, the size of the HIV reservoirs as well as immune parameters, including markers of exhaustion and activation, returned to pre-ATI levels 6–12 months after the study participants resumed ART. Of note, the proportions of near full-length, genome-intact and structurally defective HIV proviral DNA sequences were similar prior to ATI and following reinitiation of ART. In addition, there was no evidence of emergence of antiretroviral drug resistance mutations within intact HIV proviral DNA sequences following reinitiation of ART. These data demonstrate that short-term ATI does not necessarily lead to expansion of the persistent HIV reservoir nor irreparable damages to the immune system in the peripheral blood, warranting the inclusion of ATI in future clinical trials evaluating curative strategies.

## Author summary

While we have made considerable advancements in the treatment of HIV, most infected individuals require life-long treatment to suppress plasma viremia, underscoring the need for the development of additional therapeutic strategies that would allow durable virologic remission in the absence of antiretroviral therapy (ART). While a definitive cure has not yet been identified, the field is moving in a promising direction, and with continued efforts we may arrive at a clinically acceptable alternative to ART. Clinical validation of new treatment options likely requires patients to stop therapy while monitoring for viral rebound, but the effect of treatment interruption and its precise impact on immunologic and virologic parameters in HIV-infected individuals has not been fully delineated. In this work, we measured a significant increase of HIV burden in the CD4^+^ T cells of infected individuals who underwent ATI with subsequent plasma viral rebound. However, the size of the HIV reservoirs as well as immune parameters returned to pre-ATI levels 6–12 months after the participants resumed ART. These data suggest ATI does not lead to expansion of the persistent HIV reservoir nor irreversible damages to the immune system in the peripheral blood.

## Introduction

Sustained suppression of human immunodeficiency virus (HIV) and dramatic improvements in health outcomes have been achieved in infected individuals receiving antiretroviral therapy (ART) [1]. Nonetheless, the vast majority of HIV-infected individuals experience plasma viral rebound upon cessation of therapy [2], underscoring the need for developing additional therapeutic strategies that would allow durable virologic remission following the interruption of ART. Considerable efforts have been made in recent years to develop interventional approaches aimed at eliminating viral reservoirs and/or enhancing host immune responses against the virus in an effort to achieve durable suppression of HIV following discontinuation of ART [3]. In this regard, the effectiveness of such interventions has been typically evaluated *ex vivo* by measuring the impact on the size of persistent HIV reservoirs in CD4^+^ T cells of infected individuals [4]. However, these assays have proven to be inadequate for predicting whether a specific therapeutic intervention will lead to eradication of replication-competent virus or long-term suppression of HIV in the absence of ART [5–7]. Therefore, the incorporation of short-term analytical treatment interruption (ATI) into the clinical trial design has been employed to determine the efficacy of immune-based therapies in suppressing and/or eradicating HIV. Short-term ATI conducted under close virologic monitoring has been considered to be clinically safe [8]; however, its precise impact on immunologic and virologic parameters in HIV-infected individuals has not been well defined. We conducted the present study to address these issues.

## Results

### Effect of treatment interruption and subsequent resumption of ART on HIV reservoirs

**Fig 1:**
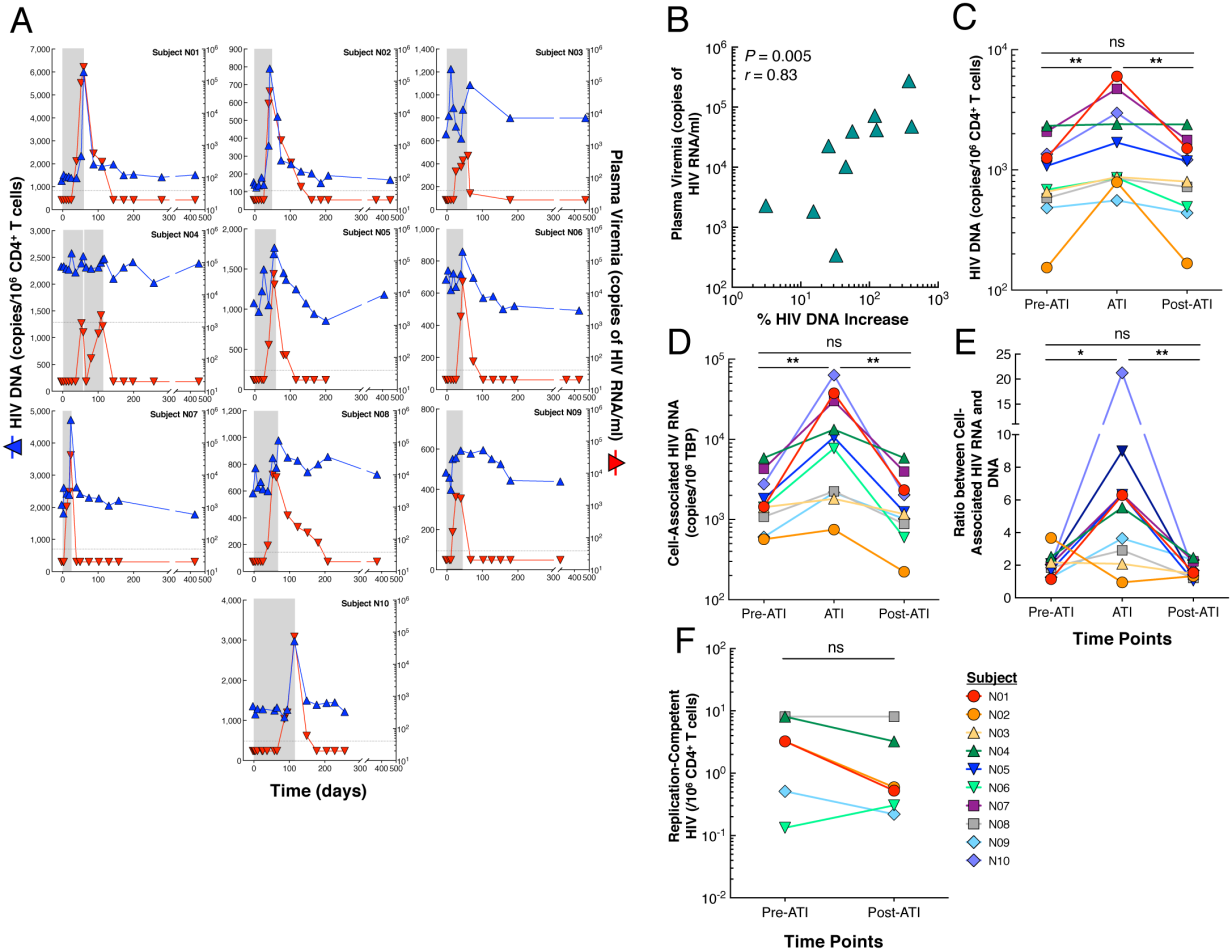
Impact of ATI and reinitiation of ART on HIV reservoirs. (**A**) Longitudinal measurements of plasma viremia (red triangles) and the frequency of CD4^+^ T cells carrying HIV DNA (blue triangles) from study participants are shown. The grey bars indicate duration of ATI. One participant (N04) self-administered antiretroviral drugs for 3 days during the ATI period. (**B**) Relationship between the level of peak plasma viremia and % increase of the frequency of CD4^+^ T cells carrying HIV DNA during the ATI phase over baseline. The % HIV DNA increase was calculated as follows: ((copy number of HIV DNA/10^6^ CD4^+^ T cells at ATI—copy number of HIV DNA/10^6^ CD4^+^ T cells at baseline)/copy number of HIV DNA/10^6^ CD4^+^ T cells at baseline)*100. (**C**) Kinetics of HIV DNA burden in CD4^+^ T cells of 10 study participants prior to ATI (Pre-ATI), during ATI (ATI), and after reinitiation of ART (Post-ATI). (**D**) Dynamics of cell-associated HIV RNA in CD4^+^ T cells of study participants prior to ATI (Pre-ATI) during ATI (ATI) and after reinitiation of ART (Post-ATI). (E) Ratios between the level of cell-associated HIV RNA and DNA. (**F**) Impact of ATI and reinitiation of ART on the level of CD4^+^ T cells carrying replication-competent HIV in 6 study participants in whom longitudinal leukapheresis was performed. Statistical significance was tested with Wilcoxon’s signed rank test for panels C, D, E, and F. A correlation was determined by the Spearman rank method for panel b. **P < 0.01, ns, not significant.

**Fig 2:**
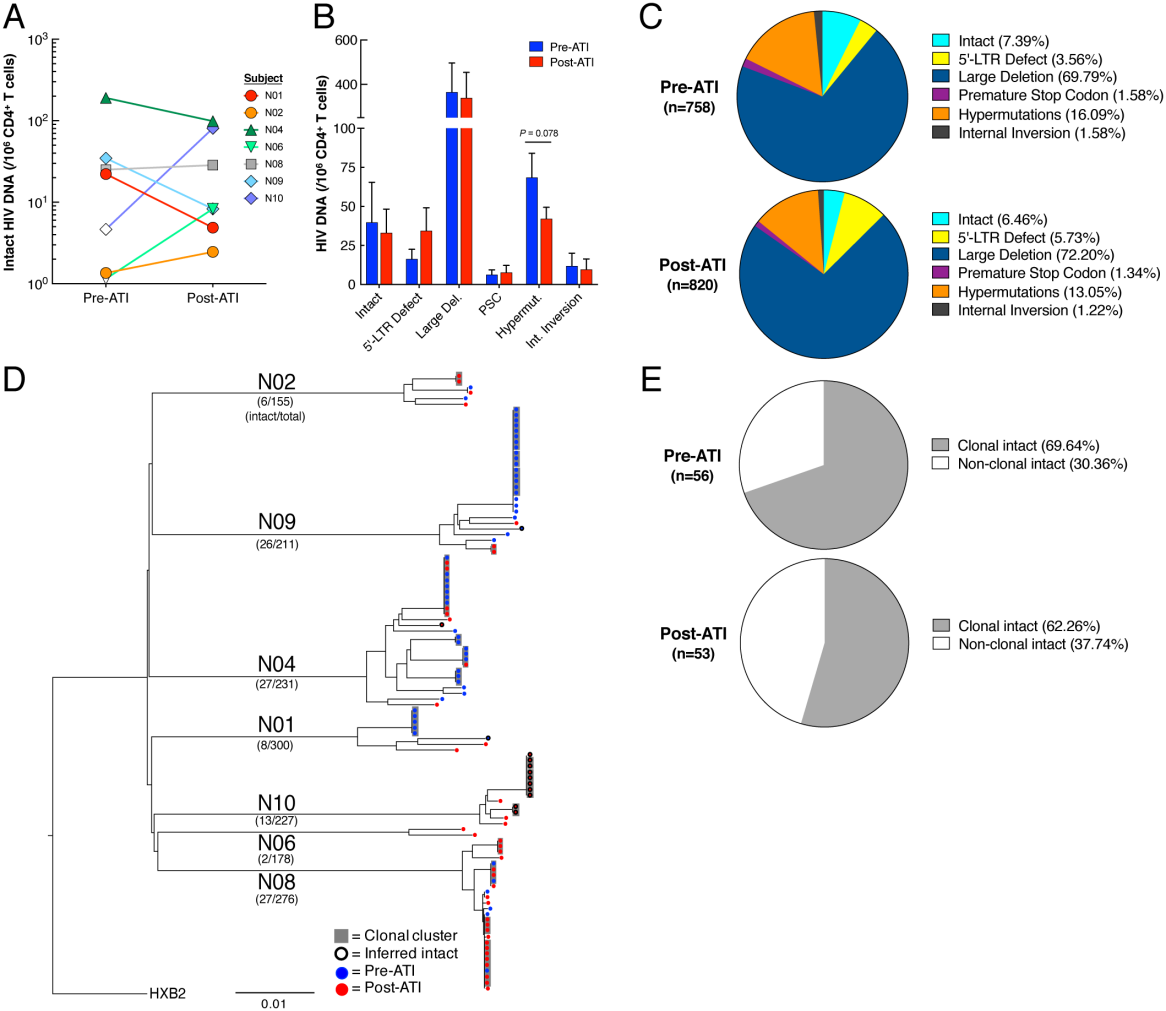
Global landscape of HIV proviral sequences prior to ATI and following reinitiation of ART. (**A**) Relative frequency of intact, near full-length HIV-1 sequences in individual study participants before and after ATI. Open symbols reflect detection threshold in cases where no intact proviruses were identified. Significance was tested using a two-tailed Wilcoxon test. (**B**) Bar diagram reflecting the relative frequency (mean and standard error) of indicated HIV-1 sequences before and after ATI. Significance was tested using a two-tailed Wilcoxon test. (**C**) Pie charts reflecting the relative contribution of proviral sequences with defined structural characteristics to the total pool of HIV-1 DNA sequences identified at the analyzed time points. (Large Del: large deletions (not sequenced), PSC: premature stop codon, Hypermut: hypermutations, Int. Inversion: internal inversion). (**D**) Phylogenetic tree summarizing all analyzed intact HIV-1 sequences. Clusters of completely identical sequences are highlighted in grey. (**E**) Pie charts indicating relative proportion of intact, clonally-expanded viral sequences before ATI and following resumption of ART.

We first investigated the effect of rebounding virus following ATI on the dynamics of HIV reservoirs using longitudinal specimens collected from 10 HIV-infected individuals who previously participated in a passive antibody transfer study (Table 1) [9]. Time points analyzed include: 1) prior to discontinuation of ART (referred to as “Pre-ATI”), 2) during ATI (referred to as “ATI”, the time point at which plasma viremia was at or closest to the highest level), and 3) following reinitiation of ART to suppress plasma viremia (referred to as “Post-ATI”). The median duration of the ATI phase was 57 days (range 22–115). All patients rebounded with observed peak viremia of 30,950 copies of HIV RNA per ml of plasma (median, mean 50,911, range 340–273,221). All study participants reinitiated ART under the pre-defined criteria of the ATI protocol and had been resumed on ART for a median of 363 days (range 140–418) post ATI at the time of analysis. Of note, one participant (N10) had received ART for 140 days when post-ATI analyses were conducted. Upon rebound, the level of total HIV DNA in the CD4^+^ T cell compartment increased significantly compared to that at the pre-ATI level (*P* = 0.002, Fig 1A–1C). There was a strong correlation between the level of plasma viremia and the percent increase of HIV DNA burden following cessation of ART (*P* = 0.005, Fig 1B). However, following reinitiation of ART, HIV DNA burden declined and subsequently reached pre-ATI levels (*P* = 0.002, Fig 1C), resulting in no statistically significant difference in HIV DNA between the pre-ATI and post-ATI time points. A similar pattern was observed when the level of cell-associated HIV RNA was measured prior to and during ATI as well as following reinitiation of ART (Fig 1D). There was a significant increase in the ratio of HIV-1 RNA to DNA during ATI compared to that at the pre-ATI level. However, following reinitiation of ART, the ratio declined and subsequently reached pre-ATI levels, resulting in no statistically significant difference in the ratio between the pre-ATI and post-ATI time points (Fig 1E). Given that the vast majority of infected CD4^+^ T cells carry replication-defective HIV, we then sought to determine the impact of ATI on the persistent viral reservoir carrying replication-competent HIV. As show in Fig 1F, there were no significant differences with respect to the number of CD4^+^ T cells carrying replication-competent HIV prior to ATI and following reinitiation of ART (*P* = 0.13). To obtain a more comprehensive view of the impact of ATI on the viral reservoir landscape, we used single-genome, near full-length viral, next-generation sequencing to assess the relative frequency of intact and defective HIV proviral sequences at the pre-ATI and post-ATI time points in subjects who underwent longitudinal leukapheresis (N01, N02, N04, N06, N08, N09, and N10). Analysis of 758 at pre-ATI (N01, n = 118; N02, n = 78; N04, n = 134; N06, n = 102; N08, n = 86; N09, n = 143; and N10 = 97) and 820 individual sequences at post-ATI (N01, n = 182; N02, n = 77; N04, n = 97; N06, n = 76; N08, n = 190; N09, n = 68; and N10, n = 130) revealed that the frequency of intact HIV proviral sequences was remarkably similar at the pre-ATI and post-ATI time points (mean of 40 vs. 33 intact proviral sequences per 10^6^ CD4^+^ T cells, respectively), as was the relative proportion of intact proviruses within the total pool of analyzed HIV DNA sequences (7.4% vs. 6.4% of all sequences, respectively) (Fig 2A–2C). In addition, the contribution of viral sequences with defined structural defects to the total pool of reservoir sequences was not significantly different between the two time points, although there was a trend toward a reduced proportion of hypermutated, near full-length sequences following reinitiation of ART (Fig 2B and 2C). Of note, clusters of intact proviral sequences that were completely identical and likely derived from clonally-expanded HIV-infected CD4^+^ T cells [10–12] accounted for 69.6% and 62.3% of all intact sequences at the pre-ATI and post-ATI time points, respectively (Fig 2D and 2E), indicating that ATI had no substantial impact on the relative proportion of clonally-expanded CD4^+^ T cells harboring intact HIV-1 (*P* = 0.42, two-tailed Fisher’s exact test). Interestingly, an analysis of antiretroviral drug-induced viral sequence polymorphisms did not show any evidence for an accumulation of known drug escape mutations after ATI in intact proviruses (S1 Table). However, intact proviral sequences at the pre-ATI and post-ATI time points displayed significant differences in the amino acid composition at several residues within recognized VRC01 contact regions, specifically at viral envelope position 363, a site known to influence sensitivity to VRC01 [13] (S1 Fig); this is consistent with the previously-described evolution of VRC01-resistant viruses during this study [9]. Collectively, our data demonstrate that the increased level of cell-associated HIV in CD4^+^ T cells during short-term treatment interruption is transient and subsequent reinitiation of ART returns the viral burden to its pre-ATI level.

**Table 1:** Profiles of HIV-infected study participants.

Subject	Race	Gender	Age	ART regimen prior to ATI*	Duration of viral suppression (years)	Nadir CD4^+^ T cell count (per mm^3^)	CD4^+^ T cell count prior to ATI (per mm^3^)	CD4^+^ T cell % prior to ATI	CD8^+^ T cell count prior to ATI (per mm^3^)	CD8^+^ T cell % prior to ATI	Plasma viremia prior to ATI (per ml)^†^	Duration of ATI (days)	Duration of ART following reinitiation of ART (days)
N01	Black	F	36	TDF, FTC, EFV	13.4	290	1,616	51	982	31	<40	59	373
N02	White	M	54	TDF, FTC, ATV/r	16.8	311	728	47	526	34	<40	46	407
N03	Black	F	33	TDF, FTC, EVG, COBI	9.2	832	1,194	47	813	32	<40	55	418
N04	White	M	59	ABC, 3TC, DTG	11.5	200	726	50	581	40	<40	114	343
N05	White	M	59	TDF, FTC, EVG, COBI	13.8	16	577	38	577	38	<40	59	353
N06	White	M	48	ABC, 3TC, DTG	7.1	581	594	32	873	47	<40	45	386
N07	Black	M	57	ABC, 3TC, DTG	3.0	NA	722	29	623	25	<40	22	409
N08	White	M	49	TDF, FTC, RPV	6.6	345	634	50	406	32	<40	67	302
N09	White	M	53	ABC, 3TC, ATV	7.5	900	992	34	1,372	47	<40	42	305
N10	White	M	43	FTC, TDF, RPV	6.7	524	628	30	1,151	55	<40	115	140
**Median**			**51**		**8.3**	**345**	**724**	**43**	**718**	**36**		**57**	**363**

*Nucleoside reverse transcriptase inhibitors: Abacavir (ABC), Emtricitabine (FTC), Lamivudine (3TC), Tenofovir (TDF); nonnucleoside reverse transcriptase inhibitors: Efavirenz (EFV), Rilpivirine (RPV); protease inhibitors: Atazanavir (ATV), Atazanavir/Ritonavir (ATV/r); integrase inhibitor: Dolutegravir (DTG), Elvitegravir (EVG); pharmacokinetic enhancer, Cobicistat (COBI).

^†^measured by Abbott Real-Time HIV-1 Assay.

NA, not available.

### Effect of treatment interruption and subsequent resumption of ART on immune parameters, gene expression, and soluble markers in plasma

**Fig 3:**
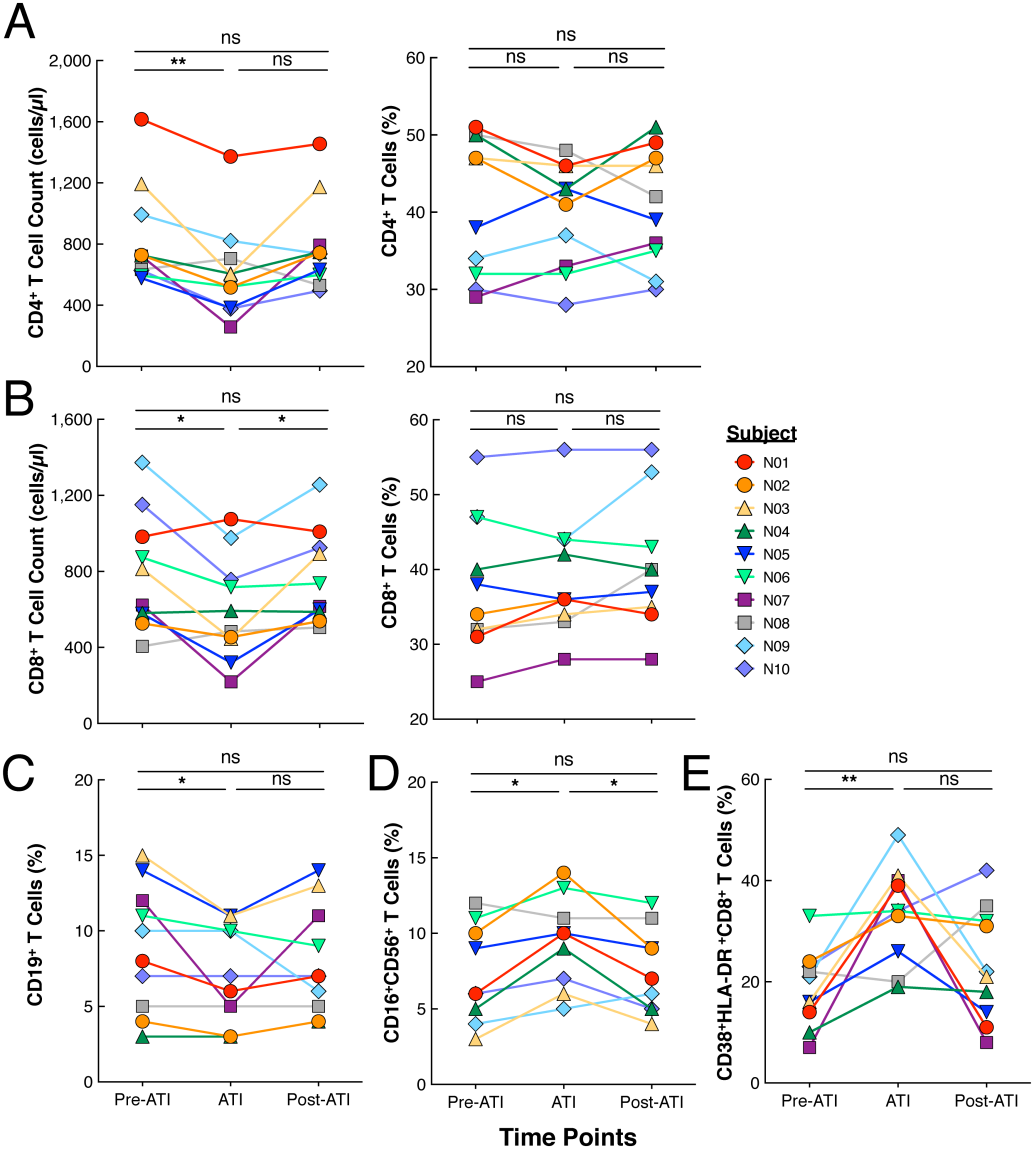
Immunologic parameters monitored for the duration of the trial. (**A**) CD4^+^ and (**B**) CD8^+^ T cell counts and percentages prior to treatment interruption (Pre-ATI), during ATI (ATI), and following reinitiation of ART (Post-ATI) and levels of (**C**) B cells, (**D**) NK cells, and (**E**) CD8^+^ T cells expressing CD38 and HLA-DR prior to treatment interruption (Pre-ATI), during ATI (ATI), and following reinitiation of ART (Post-ATI).

It is well established that active HIV replication leads to numerous immunologic abnormalities in infected individuals. In order to further investigate the effects of ATI followed by reinitiation of ART, we examined multiple immune parameters in the study participants. Although there was a transient decrease in CD4^+^ and CD8^+^ T cells during the ATI phase, there were no significant differences in the levels of CD4^+^ and CD8^+^ T cells comparing pre-ATI and post-ATI time points (Fig 3A and 3B). Additionally, there were no significant differences in the percentages of B (CD19^+^, Fig 3C), NK (CD16^+^56^+^, Fig 3D), or activated CD8^+^ T (CD38^+^HLA-DR^+^, Fig 3E) cells between pre-ATI and post-ATI time points. We also investigated the impact of ATI and reinitiation of ART on gene expression in highly purified CD4^+^ and CD8^+^ T cells (S2 Fig). A small number of genes (38 of >20,000 genes) were shown to be differentially expressed predominantly in the CD8^+^ T cells of the study participants prior to ATI and following reinitiation of ART only when unadjusted analyses were performed (S2 Fig and S2 Table). However, no significant changes in expression of genes associated with HIV life cycle and pathogenesis, interferon-regulated genes, or regulation of immune activation were found in the CD4^+^ and CD8^+^ T cells when measured prior to ATI and following reinitiation of ART (S2 Fig and S3 Table). In addition, analyses of longitudinal plasma samples revealed no change in the level of the vast majority of cytokines, chemokines, and inflammation markers between the two time points (S4 Table). Of note, a significant increase in the plasma level of RANTES following reinitiation of ART was observed (S3 Fig) although the significance of this finding requires further investigation. Collectively, our data suggest that short-term ATI and subsequent reinitiation of ART does not contribute to permanent immunologic or inflammatory abnormalities associated with the active HIV replication that results from ATI.

### Impact of treatment interruption and subsequent resumption of ART on T cell activation and exhaustion

**Fig 4:**
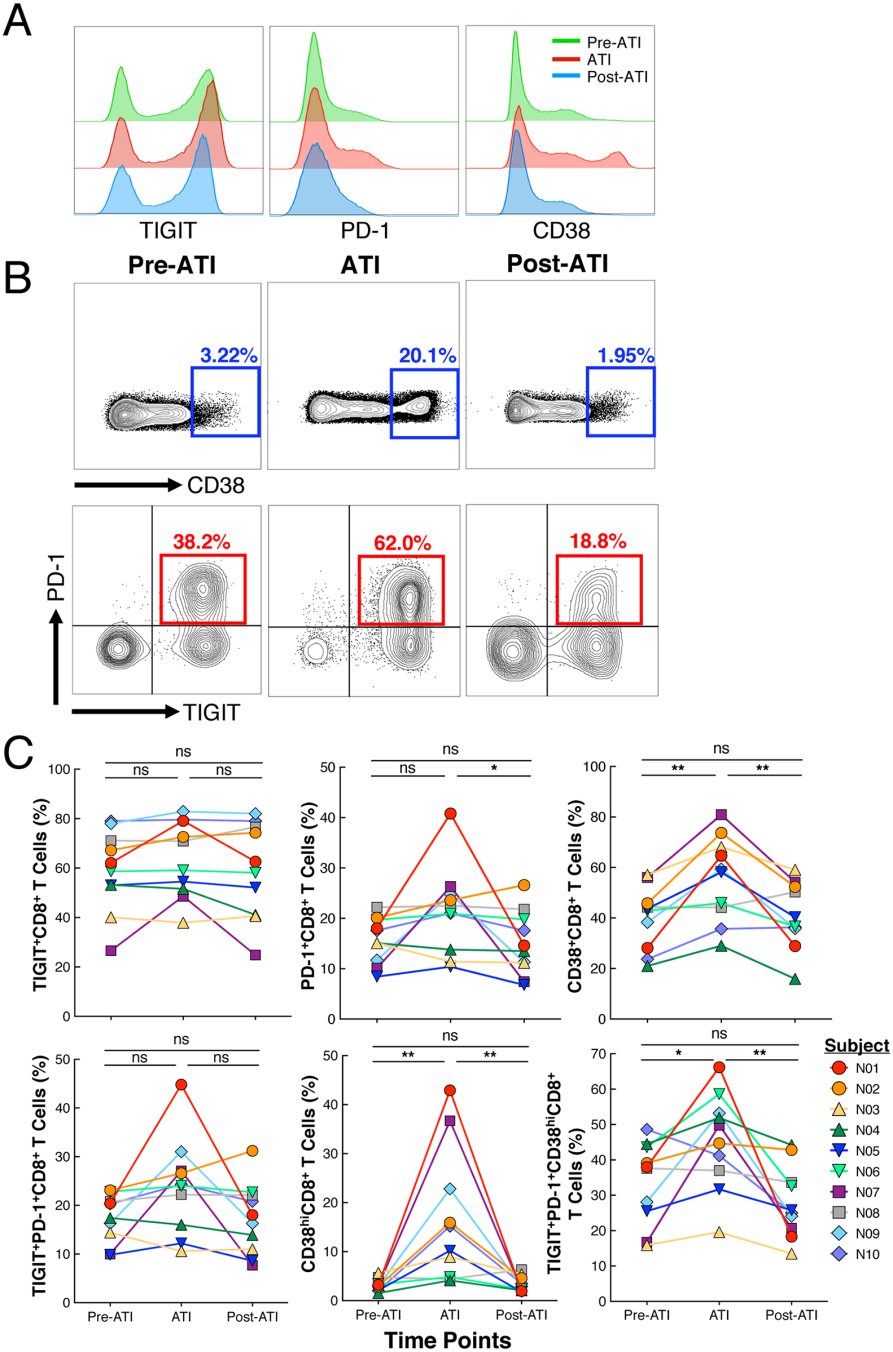
Longitudinal changes in exhaustion and activation parameters. (**A**) Longitudinal changes in expression of TIGIT, PD-1, and CD38 on CD8^+^ T cells in a representative patient at the pre-ATI, ATI, and post-ATI time points. (**B**) FACS plots showing the gating strategy on the CD38^hi^CD8^+^ T cells at the pre-ATI, ATI, and post-ATI time points. The emergence of the CD38^hi^ population during the ATI phase is exhibited in the first row. The second row demonstrates the increase in the PD-1/TIGIT double positive population within the CD38^hi^ CD8^+^ T cells during ATI followed by a subsequent decrease after reinitiation of ART. (**C**) Summary of TIGIT, PD-1, and CD38 expression on CD8^+^ T cells as well within the CD38^hi^CD8^+^ T cell subset at pre-ATI, ATI, and post-ATI time points in the first and second row, respectively, for all ten patients included in the study. Statistical significance was tested with Wilcoxon’s signed rank test. *P < 0.05, **P < 0.01, ns, not significant.

Finally, in order to further determine the functional status of T cells prior to and during the ATI as well as after reinitiation of ART, we longitudinally analyzed expression of exhaustion and activation markers on the CD8^+^ T cells of the study participants. We specifically focused on CD38^+^CD8^+^ T cells, a subset of CD8^+^ T cells that are over-represented in HIV-infected individuals during periods of active viral replication [14]. In addition, we measured the expression of immune exhaustion markers TIGIT and PD-1 on CD8^+^ T cells during the various phases of the study. These markers are expressed during chronic viral infection and are associated with decreased CD8^+^ T cell function [15]. During the short ATI phase, TIGIT expression, which is up-regulated on exhausted T cells in cancer as well as during chronic viral infection including HIV-infection [16–18], did not change significantly (Fig 4A–4C). The expression of PD-1 on CD8^+^ T cells increased in a majority of patients during the ATI phase, with the changes between the ATI phase and the post-ATI time point being statistically significant; however, the difference between pre-ATI and post-ATI was not significant (Fig 4A and 4C). Notably, the level of CD38 expression on CD8^+^ T cells elevated transiently during the ATI phase compared to that of the pre-ATI time point (*P* = 0.004) and subsequently returned to baseline during the post-ATI period (Fig 4A and 4C). Of interest, CD8^+^ T cells expressing a high level of CD38 (CD38^hi^) cells emerged in the majority of study participants during the ATI phase (Fig 4B and 4C). Further flow cytometric characterization revealed the majority of CD38^hi^CD8^+^ T cells co-expressed elevated levels of PD-1 and TIGIT during the ATI phase concomitant with plasma viral rebound that was significantly increased compared to the pre-ATI level (*P* = 0.03, Fig 4C). Following reinitiation of ART, the degree of CD38^hi^CD8^+^ T cells expressing PD-1 and TIGIT decreased significantly relative to that of the ATI phase (*P* = 0.002) and was not statistically different from the pre-ATI level (Fig 4C). Of note, the level of the above immune parameters on the CD4^+^ T cells of the study participants did not significantly change over time (S4 Fig). Taken together, these data indicate that active HIV replication/rebounding plasma viremia contributes to transient expansion of a dysfunctional subset of CD8^+^ T cells during the ATI phase that return to baseline after reinitiation of ART.

## Discussion

A major focus of current HIV research is the development of therapeutic strategies to achieve sustained virologic remission following discontinuation of ART either by eradication of viral reservoirs or enhancement of host immunity against HIV [19]. These efforts towards durable viral suppression are being driven by the fact that HIV reservoirs persist despite clinically effective treatment [20–22] and subsequent rebound occurs in virtually all infected individuals upon discontinuation of therapy [2, 23]. The efficacy of such strategies is typically assessed by laboratory-based assays *ex vivo*; however, the majority of such assays lack physiologic relevance and are unable to predict the likelihood of clinical outcome. Although the ultimate evaluation of the efficacy of a therapeutic agent in achieving sustained viral suppression would require discontinuation of ART and monitoring of plasma viremia, treatment interruption studies, especially those with repeated and prolonged cycles of ATI and infrequent monitoring, have shown adverse immunologic and virologic consequences. Despite this past experience, it remains unclear whether short-term ATI accompanied by frequent monitoring and strict ART restart criteria would have similar consequences in infected patients [8]. A thorough evaluation of this issue could potentially have a major impact on the future design of therapeutic studies in HIV-infected individuals. In the current study, we have demonstrated that short-term ATI causes transient expansion of the HIV reservoir in CD4^+^ T cells; however, the frequency of infected cells, including those carrying replication-competent HIV, returned to the pre-ATI level after reinitiation of ART. Notably, the relative frequency of near full-length viral sequences classified as genome-intact and the relative proportions of proviral sequences with defined structural defects were similar prior to ATI and following reinitiation of ART although it may require inclusion of larger copy numbers of genome-intact full-length HIV sequences obtained from a large number of participants who are not receiving interventional agents in order to validate our findings. Of note, ATI was not associated with an accumulation of intact proviral sequences that encode for antiretroviral drug resistance mutations that could potentially compromise treatment responses upon reinstitution of ART. It is not clear whether the diminution of the size of HIV reservoir following reinitiation of ART was due to decay of labile, infected cells (i.e., those with unintegrated HIV DNA) or due to death of productively infected cells. Future experiments involving a larger cohort of study participants and extensive phylogenetic analyses of subsets of CD4^+^ T cells could address this issue. Our data also demonstrate that short-term ATI was not associated with irreversible immune system abnormalities, such as a decrease in the level of CD4^+^ T cells or an increase in the markers of immune exhaustion and activation on CD8^+^ or CD4^+^ T cells. Of note, ATI/rebound was associated with emergence of CD38^+^, particularly CD38^hi^, CD8^+^ T cells. A significant proportion of these CD38^hi^ CD8^+^ T cells co-expressed TIGIT and PD-1, markers expressed on activated and exhausted cells, during the ATI phase. However, similar to what was observed with the transient increase in the HIV reservoir associated with ATI, the level of expression of these markers normalized to the pre-ATI levels following reinitiation of ART. With recent studies demonstrating that co-blockade of the TIGIT and PD-1/PD-L1 pathway is a potential target for immune restoration in HIV-infected participants [16], the blunting of emergent CD38^hi^CD8^+^ T cells co-expressing TIGIT and PD-1 could potentially contribute to better virologic suppression in the absence of ART. Finally, the expression of TIGIT and PD-1 on CD4^+^ T cells did not change significantly before, during, or after the ATI phase as shown in the supplemental data. One of the potential confounding factors of our study is that the participants received VRC01 prior to and following discontinuation of ART that may have influenced virologic and immunologic outcomes [24]. Although our data suggest VRC01 was unable to neutralize all infectious viral isolates examined, it is plausible that it may have exhibited partial antiviral effects as evidenced by emergence of antibody-resistant virus. However, it is unlikely that VRC01 had any significant impact on the parameters examined in the present study for the following reasons: 1) all study participants experienced substantial levels of plasma viral rebound (median 30,950 copies of HIV RNA/ml), comparable to that seen in a previous study [25], following discontinuation of ART accompanied by a statistically significant increase in the frequency of CD4^+^ T cells carrying both proviral HIV DNA as well as cell-associated HIV RNA that ultimately returned to baseline levels following reinitiation of ART; 2) the majority of the study participants were found to carry VRC01-resistant replication-competent HIV prior to administration of the antibody [9]; 3) following administration of VRC01, antibody-resistant HIV emerged in the majority of the study participants during the course of ATI, which led to seeding of the CD4^+^ T cell compartment with replication-competent viruses that were unable to be neutralized by VRC01 *ex vivo* [9] and exhibited features of sequence diversification at VRC01 contact residues; 4) CD8^+^ T cells that displayed dysfunctional and exhausted characteristics typically associated with active HIV replication appeared during the ATI period; and 5) the last time points measured occurred one year after reinitiation of ART, well past the half-life of the VRC01 antibody, making it highly unlikely to be a source of virologic and immunological influence. Further investigations addressing longitudinal examination of tissue compartments and meta-analytic explorations may be necessary to formally evaluate the impact of ATI and re-initiation of ART on the size of the persistent HIV reservoir as well as immune parameters in a larger cohort of study participants who are not receiving other investigational anti-HIV agents (i.e., broadly neutralizing HIV-specific antibodies). Nonetheless, our findings support the use of antiretroviral treatment interruption in the setting of close monitoring of plasma viremia and concomitant strict ART restart guidelines as an integral part of determining the *in vivo* efficacy of therapeutic strategies aimed at achieving sustained ART-free virologic suppression in HIV-infected individuals.

## Materials and methods

### Ethics statement

Blood and leukapheresed products were collected from the study participants in accordance with clinical protocols approved by the Institutional Review Boards of the National Institute of Allergy and Infectious Diseases at the National Institutes of Health. All study participants were adults and provided written informed consent. All samples were anonymized.

### Study participants

Ten HIV-infected individuals from the NIH cohort (Participants N01-N10) who previously participated in a passive antibody transfer study (VRC01) were studied (Table 1). Participants received infusions of VRC01 (40mg/kg) intravenously three days before and 14 and 28 days after the discontinuation of ART followed by monthly thereafter for up to 6 months. Participants who met any of the following criteria discontinued VRC01 infusions and resumed ART: a decrease of more than 30% in the baseline CD4 T-cell count or an absolute CD4 T-cell count below 350 cells per cubic millimeter, a sustained (≥2 weeks) HIV plasma viremia greater than 1,000 copies per milliliter, any HIV-related symptoms, or pregnancy [9].

### Measurements of HIV DNA in CD4^+^ T cells

In order to measure the frequency of cells carrying HIV DNA, genomic DNA was isolated from purified CD4^+^ T cells using the Puregene DNA Extraction kit (Qiagen). The extracted DNA was digested with MscI (New England BioLabs) and subjected to droplet digital PCR (Bio-Rad Laboratories) according to the manufacturer’s specifications. The PCR reaction was carried out using HIV-specific and housekeeping gene RPP30-specific primers and probes in triplicate. The following primers were used for amplification of HIV LTR: 5’- GRAACCCACTGCTTAAGCCTCAA -3’ (5’ primer) and 5’- TGTTCGGGCGCCACTGCTAGAGA -3’ (3’ primer) along with the fluorescent probe 5’-6FAM-AGTAGTGTGTGCCCGTCTGTT-IABkFQ-3’. The following primers were used for amplification of RPP30: 5’-GATTTGGACCTGCGAGCG-3’ (5’ primer) and 5’-GCGGCTGTCTCCACAAGT-3’ (3’ primer) along with the fluorescent probe 5’-HEX-TTCTGACCTGAAGGCTCTGCGC-IABkFQ-3’.

### Quantitation of cell-associated HIV RNA

In order to determine the level of cell-associated HIV RNA, RT-PCR was carried out using RNA isolated from purified CD4^+^ T cells (RNeasy Mini kit, Qiagen). Subsequently cDNA was synthesized from 2μg of RNA using qScript XLT cDNA Master Mix (Quanta Biosciences) using the following incubation steps: 5 minutes at 35°C, 60 minutes at 42°C, and 5 minutes at 85°C. cDNA was then subjected to droplet digital PCR (Bio-Rad) using HIV-specific and housekeeping gene, TATA box binding protein (TBP)-specific primers and probes in triplicate. The following primers were used for amplification of HIV 5’- TCTCTAGCAGTGGCGCCCGAACA -3’ (5’ primer) and 5’- TCTCCTTCTAGCCTCCGCTAGTC -3’ (3’ primer) along with the fluorescent probe 5’-6FAM- CAAGCCGAGTCCTGCGTCGAGAG -IABkFQ-3’. The following primers were used for amplification of TBP: 5’- CACGAACCACGGCACTGATT -3’ (5’ primer) and 5’- TTTTCTTGCTGCCAGTCTGGAC -3’ (3’ primer) along with the fluorescent probe 5’-HEX- TGTGCACAGGAGCCAAGAGTGAAGA/3-IABkFQ-3’. HIV RNA copy numbers were normalized per 1x10^6^ copies of TBP.

### Measurements of replication-competent HIV

In order to determine the level of total CD4^+^ T cells carrying replication-competent/infectious virus, serially diluted (1x10^6^, 200,000, 40,000, 8,000, 1,600, 320) and replicates of 5x10^6^ CD4^+^ T cells were subjected to quantitative co-culture assays. The cultures were then incubated with irradiated PBMCs (6–8 x10^6^ cells per well) obtained from healthy sero-negative donors and anti-CD3 antibody. 1x10^6^ CD8-depleted and anti-CD3 stimulated PBMCs from HIV-negative donors were added to each well the following day followed by periodic removal of cell suspensions and replenishment with fresh media containing IL-2. HIV p24 ELISA was conducted on the culture supernatants between days 14 and 21. The infectious units per million cells (IUPM) from quantitative co-culture assays were determined as described [26].

### Viral sequencing

Genomic DNA was extracted from highly enriched CD4^+^ T cells using the QIAGEN DNeasy Blood & Tissue kit (Qiagen). HIV-1 gag DNA was then quantified using digital-droplet PCR (ddPCR). DNA diluted to single viral genome levels (<25% of wells being positive for HIV DNA PCR products) based on Poisson distribution statistics and ddPCR results was subjected to amplification using Platinum Taq DNA polymerase (ThermoFisher Scientific) and nested primers [12] spanning near full-length HIV-1 (HXB2 coordinates 638–9632). PCR products were visualized by agarose gel electrophoresis. Near full-length sequences (>8000 bp) were subjected to Illumina MiSeq sequencing with a median of approximately 2500 reads per base. Resulting short reads were *de novo* assembled and aligned to HXB2 using MUSCLE [27] to identify premature/lethal stop codons, internal inversions, or packaging signal deletions, using an automated in-house pipeline written in R scripting language that analyzes all open reading frames [28]. Presence/absence of APOBEC-3G/3F-associated hypermutations was determined using the Los Alamos HIV Sequence Database Hypermut 2.0 program [29]. Viral sequences that lacked all mutations listed above and had less than 15 base pairs deletions in the sequenced 5-LTR region were classified as “intact” [30]. If a near-full length sequence showed a mapped 5’ LTR deletion with an absent or incomplete primer binding site, but otherwise displayed no lethal sequence defects, the missing 5’ LTR sequence was inferred to be present, as described elsewhere [30] and the sequence was termed “inferred-intact”. Sequences identified as intact or inferred-intact by this algorithm were selected for manual verification of all open reading frames [12]. Phylogenetic distances between sequences were examined using ClustalX-generated neighbor joining algorithms [31]. The assay was validated by repeated (50 consecutive times) single genome amplification and sequencing of near full-length HIV DNA from the 8E5 cell line, which resulted in completely identical sequences in each case. Sequencing products with two different viral contigs, which would suggest amplification of two different viral DNA products in the same well, were discarded and not included in the analysis.

### Flow cytometry

Peripheral blood mononuclear cells (PBMC) were isolated from peripheral blood and leukapheresis by Ficoll-Hypaque density gradient centrifugation and cryopreserved in liquid nitrogen. Cryopreserved PBMCs were thawed, washed, and stained with the following fluorophore-conjugated antibodies: CD3-APC-H7 (clone SK7, BD#560176), CD4-APC (clone SK3, BD#340443), CD38-BV421 (clone HIT2, BD#562444), TIGIT-PE (clone MBSA43, ebioscience#12–9500), PD-1- PE-Cy7 (clone eBioJ105, ebioscience#25–2799), LAG-3-PerCP-eF710 (clone 3DS223H, ebioscience#46–2239), TIM-3-FITC (clone F38-2E2, ebioscience#11–3109). Data were acquired on a BD FACSCanto II flow cytometer using the FACSDiva software (Becton Dickinson) and analyzed using Flow Jo version 10.1r5. Flow cytometry was repeated on specimens from select patients to ensure reproducibility.

### Microarray analysis

RNA from CD4^+^ and CD8^+^ T cells was isolated using RNeasy mini kit (Qiagen) according to manufacturer’s specifications with on-column DNase I digestion to remove genomic DNA. 100 ng of total RNA was amplified and labeled using GeneChip 3’IVT PLUS Reagent Kit (Affymetrix), according to manufacturer’s instructions. 6μg of labeled cRNA was hybridized for 16h in 45°C to Affymetrix Human Genome U219 Array Strip, which contains more than 530,000 probes covering more than 36,000 transcripts and variants, which represent more than 20,000 genes mapped through UniGene or via RefSeq annotation. The arrays were washed and stained using GeneAtlas Hybridization, Wash, and Stain Kit for 3’IVT Arrays. CEL files retrieved from the GeneAtlas Software were normalized by Robust Multiarray Average (RMA), and further analyzed for differential gene expression by one-way analysis of variance (ANOVA), using Partek Genomics Suit 6.6.

### Quantification of cytokines and inflammation markers in plasma

Levels of 61 cytokines and chemokines (Human Cytokine Group I kit, Bio-Rad) and inflammation markers (Human Inflammation Panel I kit, Bio-Rad) were measured in undiluted plasma samples collected from the study participants prior to ATI and following reinitiation of ART according to the manufacturer’s instructions. Immunoassays were performed using the Bio-Plex 200 instrument (Bio-Rad) and analyzed with the Bio-Plex Manager software (Bio-Rad) using standard curves generated from the provided recombinant standards.

### Statistical analysis

Three-way comparisons were performed using the Friedman test followed by pair-wise comparisons with the Wilcoxon signed rank test if significant. Correlations were determined by the Spearman rank method. The statistical tests used for each experiment are indicated in the figure legends. *P* < 0.05 was considered significant.

## Supporting information

S1 Fig Amino acid composition in VRC01 contact regions within intact HIV proviral DNA sequences detected before and after ATI. One hundred nine intact full-length HIV proviral DNA sequences (56 pre- and 53 post-ATI) were analyzed. Sequence logos were generated with *weblogo* software. # nominal *P* < 0.05, in at least one study participant, two-tailed Chi-Square test.
(PDF)

S2 Fig Effect of ATI and reinitiation of ART on gene expression in CD4^+^ and CD8^+^ T cells of study participants. Microarray analysis was performed to evaluate changes in gene expression in highly purified CD4^+^ (top panel) and CD8^+^ (bottom panel) T cells of study participants at pre-ATI and post-ATI time points. Volcano plots depicting statistical significance (y-axis) vs. fold change (x-axis) between pre-ATI and post-ATI time point are shown for all genes tested on microarray, as well as three additional groups of genes, relevant to HIV infection and immune response[32,33]. The complete list of relevant genes is in S2 Table. The cutoff lines (red lines) are determined by the fold change threshold of 1.2 and unadjusted p-value < 0.05. No significant changes in gene expression were observed in CD4^+^ and CD8^+^ T cells between the pre-ATI and post-ATI time points.
(PDF)

S3 Fig Effect of treatment interruption and resumption of ART on levels of RANTES in plasma. The level of RANTES in plasma of the study participants were compared between the pre-ATI and post-ATI time points. A two-tailed Wilcoxon matched-pairs signed rank test was performed to obtain the p value.
(PDF)

S4 Fig Changes in exhaustion and activation parameters in CD4^+^ T cells. Levels of TIGIT, PD-1, CD38, and TIGIT^+^PD-1^+^ on CD4^+^ T cells at pre-ATI, ATI, and post-ATI time points. Statistical significance was tested with Wilcoxon’s signed rank test for panels a and d. *P < 0.05, **P < 0.01, ns, not significant.
(PDF)

S1 Table Profiles of potential antiretroviral drug escape mutations in intact HIV proviral DNA. (PDF)

S2 Table List of differentially expressed genes examined by microarray. (PDF)

S3 Table List of genes examined by microarray. (PDF)

S4 Table Profiles of soluble markers in patient plasma samples were compared between the pre-ATI and post-ATI time points. A two-tailed Wilcoxon matched-pairs signed rank test was performed with P values < 0.05 considered significant.
(PDF)
