# Supplementary figures and images for: Effect of analytical treatment interruption and reinitiation of antiretroviral therapy on HIV reservoirs and immunologic parameters in infected individuals
Source: PLoS Pathog. 2018 Jan 11;14(1):e1006792. doi: 10.1371/journal.ppat.1006792 (PMC5764487; doi:10.1371/journal.ppat.1006792)

Loop D  
(HXB2 *env* 275-283)

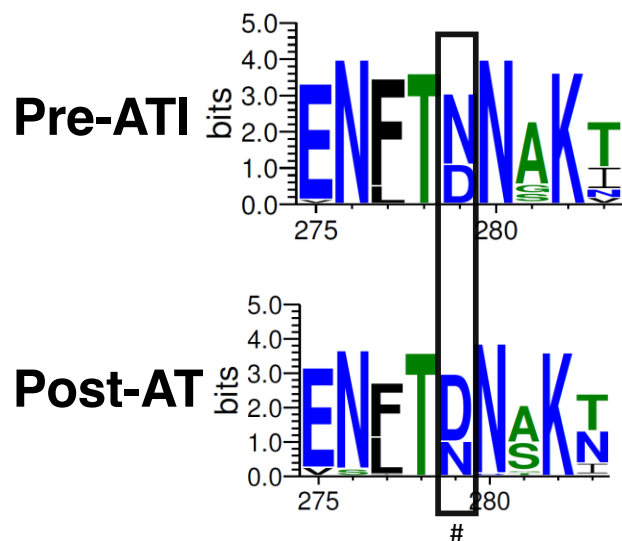

CD4 binding loop  
(HXB2 *env* 362-374)

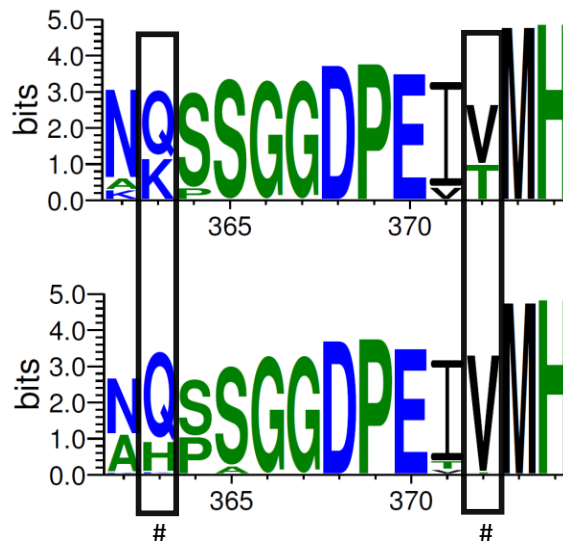

$\beta$ 23 loop V5  
(HXB2 *env* 455-467)

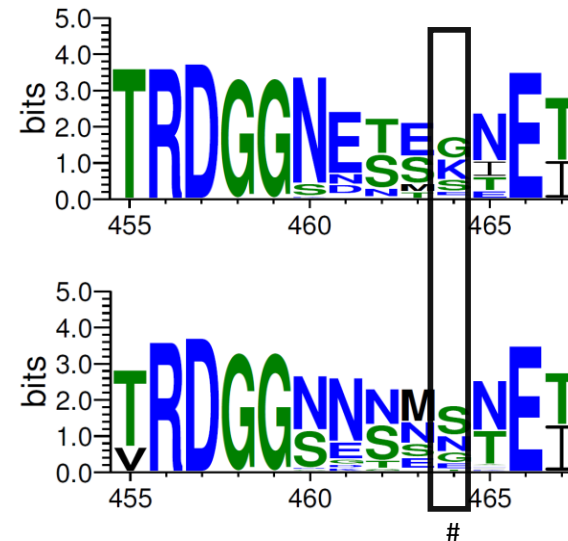

Supplement: S1 Fig — One hundred nine intact full-length HIV proviral DNA sequences (56 pre- and 53 post-ATI) were analyzed. Sequence logos were generated with weblogo software. # nominal P < 0.05, in at least one study participant, two-tailed Chi-Square test. (PDF) [file ppat.1006792.s001.pdf]

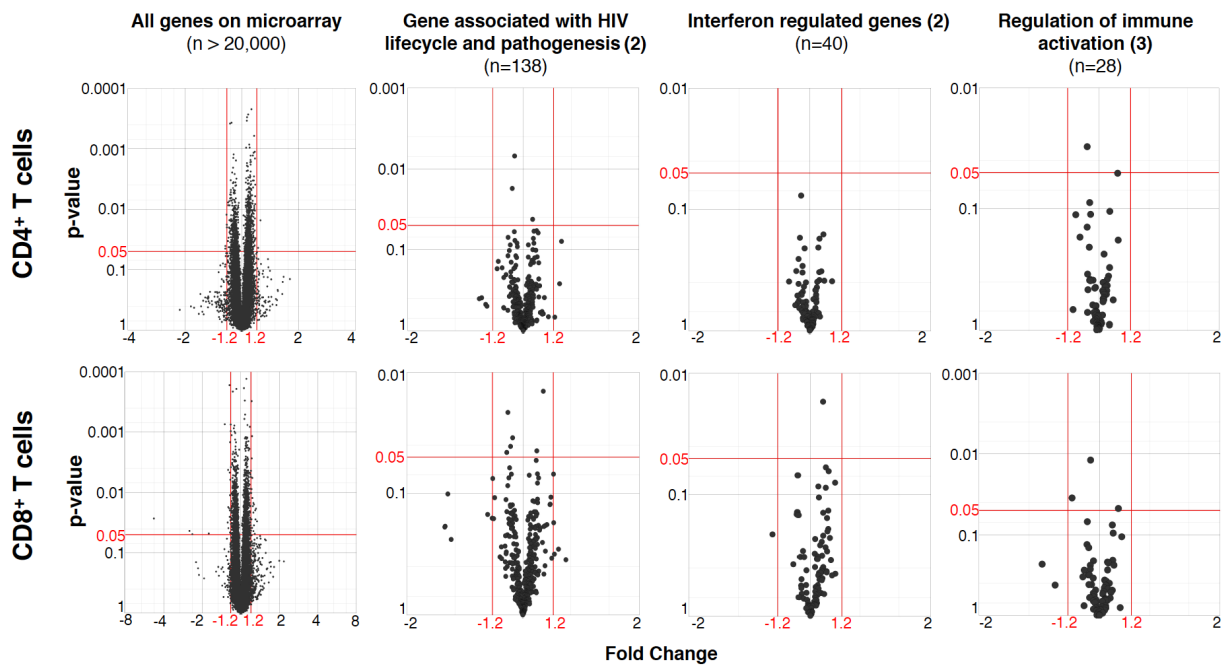

Supplement: S2 Fig — Microarray analysis was performed to evaluate changes in gene expression in highly purified CD4+ (top panel) and CD8+ (bottom panel) T cells of study participants at pre-ATI and post-ATI time points. Volcano plots depicting statistical significance (y-axis) vs. fold change (x-axis) between pre-ATI and post-ATI time point are shown for all genes tested on microarray, as well as three additional groups of genes, relevant to HIV infection and immune response[32,33]. The complete list of relevant genes is in S2 Table. The cutoff lines (red lines) are determined by the fold change threshold of 1.2 and unadjusted p-value < 0.05. No significant changes in gene expression were observed in CD4+ and CD8+ T cells between the pre-ATI and post-ATI time points. (PDF) [file ppat.1006792.s002.pdf]

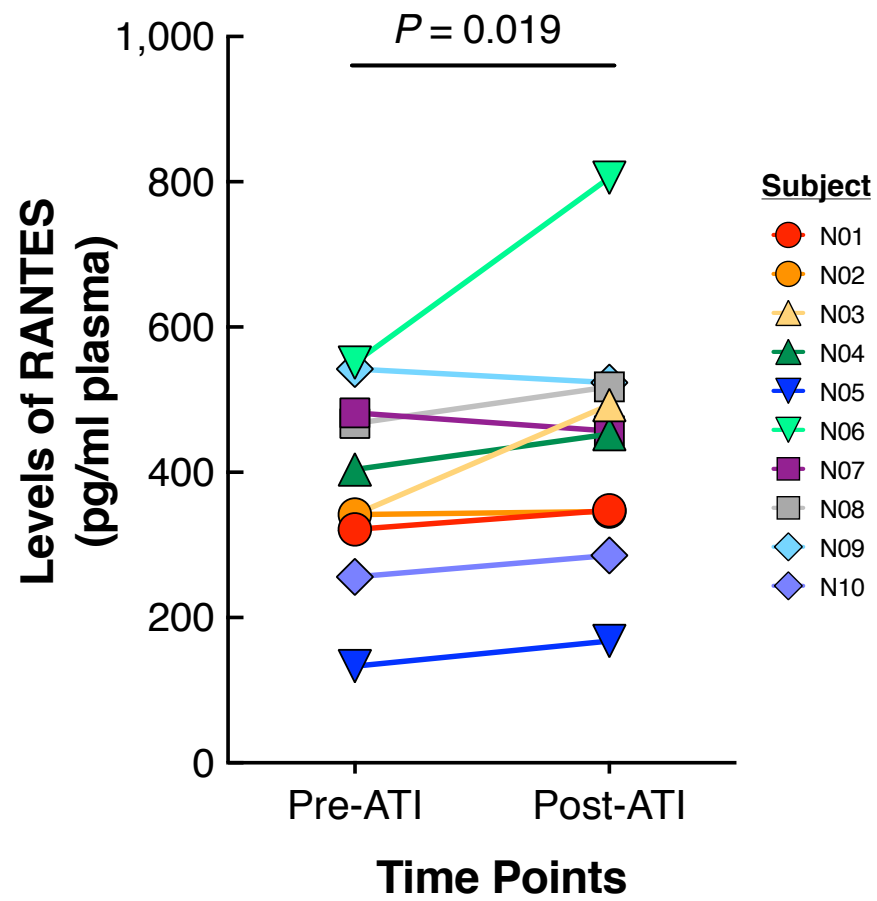

Supplement: S3 Fig — The level of RANTES in plasma of the study participants were compared between the pre-ATI and post-ATI time points. A two-tailed Wilcoxon matched-pairs signed rank test was performed to obtain the p value. (PDF) [file ppat.1006792.s003.pdf]

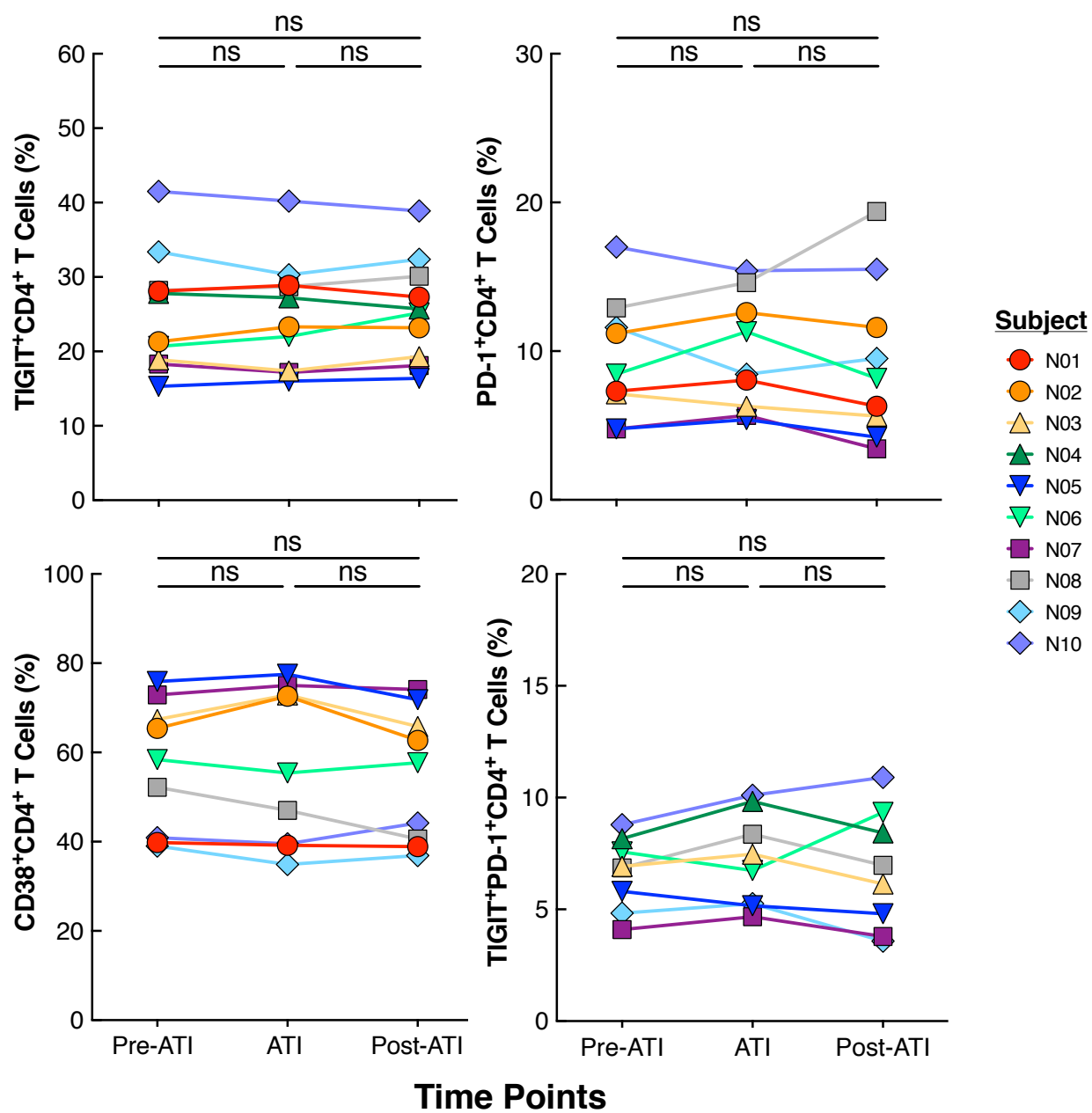

Supplement: S4 Fig — Levels of TIGIT, PD-1, CD38, and TIGIT+PD-1+ on CD4+ T cells at pre-ATI, ATI, and post-ATI time points. Statistical significance was tested with Wilcoxon’s signed rank test for panels a and d. *P < 0.05, **P < 0.01, ns, not significant. (PDF) [file ppat.1006792.s004.pdf]
