# Supplementary material for: Effect of analytical treatment interruption and reinitiation of antiretroviral therapy on HIV reservoirs and immunologic parameters in infected individuals
Source: PLoS Pathog. 2018 Jan 11;14(1):e1006792. doi: 10.1371/journal.ppat.1006792 (PMC5764487; doi:10.1371/journal.ppat.1006792)
Supplement: S1 Table — (PDF) [file ppat.1006792.s005.pdf]

S1 Table.

Sequence ID	Study Participant	Time point	Subtype	Potential Drug Escape Mutations			
				PI	NRTI	NNRTI	INI
K0004	N01	Pre-ATI	B	None	None	None	None
K0006	N01	Pre-ATI	B	None	None	None	None
K0068	N01	Pre-ATI	B	None	None	None	None
K0072	N01	Pre-ATI	B	None	None	None	None
K0080	N01	Pre-ATI	B	None	None	None	None
K0088	N01	Pre-ATI	B	None	None	None	None
K0092	N01	Post-ATI	B	None	None	None	None
K0109	N01	Post-ATI	B	None	**K70R,M184V**	None	None
K0128	N02	Pre-ATI	B	None	None	None	None
K0132	N02	Pre-ATI	B	None	None	None	None
K0180	N02	Post-ATI	B	None	None	None	None
K0181	N02	Post-ATI	B	None	None	None	None
K0017	N02	Post-ATI	B	None	None	None	None
K0189	N02	Post-ATI	B	None	None	None	None
K0020	N04	Pre-ATI	B	None	None	None	None
K0022	N04	Pre-ATI	B	None	None	None	None
K0024	N04	Pre-ATI	B	None	None	None	None
K0027	N04	Pre-ATI	B	None	None	None	None
K0028	N04	Pre-ATI	B	None	None	None	None
K0135	N04	Pre-ATI	B	None	None	None	None
K0141	N04	Pre-ATI	B	None	None	None	None
K0145	N04	Pre-ATI	B	None	None	None	None
K0146	N04	Pre-ATI	B	None	None	None	None
K0148	N04	Pre-ATI	B	None	None	None	None
K0150	N04	Pre-ATI	B	None	None	None	None
K0154	N04	Pre-ATI	B	None	None	None	None
K0163	N04	Pre-ATI	B	None	None	None	None
K0166	N04	Pre-ATI	B	None	**K65R**	None	None
K0167	N04	Pre-ATI	B	None	None	None	None
K0168	N04	Pre-ATI	B	None	None	None	None
K0169	N04	Pre-ATI	B	None	None	None	None
K0171	N04	Pre-ATI	B	None	None	None	None
K0172	N04	Pre-ATI	B	None	None	None	None
K0030	N04	Post-ATI	B	None	None	None	None
K0033	N04	Post-ATI	B	None	None	None	None
K0196	N04	Post-ATI	B	**M46I**	None	None	None
K0201	N04	Post-ATI	B	None	None	None	None
K0203	N04	Post-ATI	B	None	None	None	None
K0207	N04	Post-ATI	B	None	None	None	None
K0209	N04	Post-ATI	B	None	None	None	None
K0211	N04	Post-ATI	B	None	None	None	None
K0269	N06	Post-ATI	B	None	None	None	None
K0271	N06	Post-ATI	B	None	None	None	None
K0386	N08	Pre-ATI	B	None	None	None	None
K0289	N08	Pre-ATI	B	None	None	None	None
K0291	N08	Pre-ATI	B	None	None	None	None
K0296	N08	Pre-ATI	B	None	None	None	None
K0306	N08	Pre-ATI	B	None	None	None	None
K0311	N08	Pre-ATI	B	None	None	None	None
K0324	N08	Post-ATI	B	None	None	None	None
K0326	N08	Post-ATI	B	None	None	None	None
K0330	N08	Post-ATI	B	None	None	None	None
K0331	N08	Post-ATI	B	None	None	None	None
K0338	N08	Post-ATI	B	None	None	None	None
K0340	N08	Post-ATI	B	None	None	None	None
K0346	N08	Post-ATI	B	None	None	None	None
K0347	N08	Post-ATI	B	None	None	None	None
K0349	N08	Post-ATI	B	None	None	None	None
K0352	N08	Post-ATI	B	None	None	None	None
K0354	N08	Post-ATI	B	None	None	None	None
K0360	N08	Post-ATI	B	None	None	None	None
K0366	N08	Post-ATI	B	None	None	None	None
K0367	N08	Post-ATI	B	None	None	None	None
K0370	N08	Post-ATI	B	None	None	None	None
K0378	N08	Post-ATI	B	None	None	None	None
K0380	N08	Post-ATI	B	None	None	None	None
K0381	N08	Post-ATI	B	None	None	None	None
K0382	N08	Post-ATI	B	None	None	None	None
K0383	N08	Post-ATI	B	None	None	None	None
K0385	N08	Post-ATI	B	None	None	None	None
K0046	N09	Pre-ATI	B	None	None	None	None
K0052	N09	Pre-ATI	B	None	None	None	None
K0402	N09	Pre-ATI	B	None	None	None	None
K0404	N09	Pre-ATI	B	None	None	None	None
K0408	N09	Pre-ATI	B	None	None	None	None
K0409	N09	Pre-ATI	B	None	None	None	None
K0411	N09	Pre-ATI	B	None	None	None	None
K0412	N09	Pre-ATI	B	None	None	None	None
K0413	N09	Pre-ATI	B	None	None	None	None
K0414	N09	Pre-ATI	B	None	None	None	None
K0415	N09	Pre-ATI	B	None	None	None	None
K0417	N09	Pre-ATI	B	None	None	None	None
K0418	N09	Pre-ATI	B	None	None	None	None
K0419	N09	Pre-ATI	B	None	None	None	None
K0420	N09	Pre-ATI	B	None	None	None	None
K0421	N09	Pre-ATI	B	None	None	None	None
K0430	N09	Pre-ATI	B	None	None	None	None
K0433	N09	Pre-ATI	B	None	None	None	None
K0437	N09	Pre-ATI	B	None	None	None	None
K0446	N09	Pre-ATI	B	None	None	None	None
K0449	N09	Pre-ATI	B	None	None	None	None
K0454	N09	Pre-ATI	B	None	None	None	None
K0455	N09	Pre-ATI	B	None	None	None	None
K0470	N09	Post-ATI	B	None	None	None	None
K0474	N09	Post-ATI	B	None	None	None	None
K0496	N09	Post-ATI	B	None	None	None	None
K0059	N10	Post-ATI	B	None	None	None	None
K0060	N10	Post-ATI	B	None	None	None	None
K0062	N10	Post-ATI	B	None	None	None	None
K0514	N10	Post-ATI	B	None	None	None	None
K0521	N10	Post-ATI	B	None	None	None	None
K0522	N10	Post-ATI	B	None	None	None	None
K0525	N10	Post-ATI	B	None	None	None	None
K0527	N10	Post-ATI	B	None	None	None	None
K0528	N10	Post-ATI	B	None	None	None	None
K0530	N10	Post-ATI	B	None	None	None	None
K0535	N10	Post-ATI	B	None	None	None	None
K0536	N10	Post-ATI	B	None	None	None	None
K0537	N10	Post-ATI	B	None	None	None	None

PI, protease inhibitor, NRTI, nucleoside reverse transcriptase inhibitor, NNRTI, non-nucleoside reverse transcriptase inhibitor, INI, integrase inhibitor.
