# Supplementary material for: Effect of analytical treatment interruption and reinitiation of antiretroviral therapy on HIV reservoirs and immunologic parameters in infected individuals
Source: PLoS Pathog. 2018 Jan 11;14(1):e1006792. doi: 10.1371/journal.ppat.1006792 (PMC5764487; doi:10.1371/journal.ppat.1006792)
Supplement: S2 Table — (PDF) [file ppat.1006792.s006.pdf]

**S2 Table**.

| Gene ID | Gene Symbol | Gene Title | P value | Fold change | Note |
|---|---|---|---|---|---|
| 11715347_s_at | HBB | hemoglobin, beta | 0.0270 | -4.7874 | Upregulated at post-ATI |
| 11753712_x_at | HBB | hemoglobin, beta | 0.0434 | -2.5215 | |
| 11715348_x_at | HBB | hemoglobin, beta | 0.0491 | -2.3958 | |
| 11754267_x_at | HBA1 /// HBA2 | hemoglobin, alpha 1 /// hemoglobin, alpha 2 | 0.0480 | -1.7826 | |
| 11745049_a_at | CDC45 | cell division cycle 45 | 0.0007 | -1.3346 | |
| 11726725_a_at | NABP1 | nucleic acid binding protein 1 | 0.0315 | -1.2687 | |
| 11717805_a_at | CASP3 | caspase 3 | 0.0042 | -1.2575 | |
| 11719839_a_at | CASP3 | caspase 3 | 0.0175 | -1.2551 | |
| 11752964_a_at | SLC25A20 | solute carrier family 25 (carnitine/acylcarnitine translocase), member 20 | 0.0390 | -1.2507 | |
| 11759088_at | MARCH1 | membrane associated ring finger 1 | 0.0183 | -1.2350 | |
| 11755762_a_at | TMEM2 | transmembrane protein 2 | 0.0002 | -1.2274 | |
| 11738121_a_at | SNX18 | sorting nexin 18 | 0.0254 | -1.2231 | |
| 11719714_at | PPM1B | protein phosphatase, Mg2+/Mn2+ dependent, 1B | 0.0047 | -1.2215 | |
| 11759630_at | GPATCH2 | G-patch domain containing 2 | 0.0198 | -1.2184 | |
| 11746173_a_at | DDHD1 | DDHD domain containing 1 | 0.0111 | -1.2150 | |
| 11736328_s_at | ZNF322 | zinc finger protein 322 | 0.0460 | -1.2133 | |
| 11720367_a_at | TMEM2 | transmembrane protein 2 | 0.0015 | -1.2132 | |
| 11757720_s_at | EVPLL | envoplakin-like | 0.0062 | -1.2081 | |
| 11739934_a_at | SLC39A12 | solute carrier family 39 (zinc transporter), member 12 | 0.0256 | -1.2081 | |
| 11723336_a_at | GMIP | GEM interacting protein | 0.0290 | -1.2049 | |
| 11752279_s_at | TSPYL2 | TSPY-like 2 | 0.0044 | 1.2001 | Downregulated at post-ATI |
| 11741197_s_at | KIF5C | kinesin family member 5C | 0.0430 | 1.2010 | |
| 11747572_a_at | TMPRSS4 | transmembrane protease, serine 4 | 0.0102 | 1.2063 | |
| 11733500_a_at | ZBTB25 | zinc finger and BTB domain containing 25 | 0.0279 | 1.2110 | |
| 11726170_at | FLT4 | fms-related tyrosine kinase 4 | 0.0088 | 1.2127 | |
| 11734036_a_at | SAXO1 | stabilizer of axonemal microtubules 1 | 0.0003 | 1.2146 | |
| 11758908_a_at | ZNF827 | zinc finger protein 827 | 0.0027 | 1.2176 | |
| 11726850_a_at | ZC3H3 | zinc finger CCCH-type containing 3 | 0.0012 | 1.2236 | |
| 11764058_at | PRNP | prion protein | 0.0387 | 1.2253 | |
| 11717666_a_at | TSPYL4 | TSPY-like 4 | 0.0057 | 1.2298 | |
| 11719524_a_at | GATSL3 | GATS protein-like 3 | 0.0124 | 1.2311 | |
| 11717326_at | KLF9 | Kruppel-like factor 9 | 0.0169 | 1.2421 | |
| 11754941_x_at | KRT18 | keratin 18, type I | 0.0149 | 1.2535 | |
| 11759550_at | ZFAS1 | ZNFX1 antisense RNA 1 | 0.0215 | 1.2539 | |
| 11756387_x_at | ARL4A | ADP-ribosylation factor like GTPase 4A | 0.0438 | 1.2547 | |
| 11762321_s_at | TRAV12-2 /// YME1L1 | T cell receptor alpha variable 12-2 /// YME1-like 1 ATPase | 0.0351 | 1.2671 | |
| 11735924_s_at | KRTAP12-1 /// KRTAP12-2 | keratin associated protein 12-1 /// keratin associated protein 12-2 | 0.0158 | 1.2676 | |
| 11727569_at | OTULIN | OTU deubiquitinase with linear linkage specificity | 0.0486 | 1.2851 | |
| 11733371_at | INADL | InaD-like (Drosophila) | 0.0198 | 1.2955 | |
| 11746623_a_at | NR3C2 | nuclear receptor subfamily 3, group C, member 2 | 0.0280 | 1.3023 | |
| 11718949_a_at | MPP5 | membrane protein, palmitoylated 5 | 0.0186 | 1.3141 | |
| 11757155_x_at | SNORA3B | small nucleolar RNA, H/ACA box 3B | 0.0301 | 1.3381 | |
