# Supplementary material for: Effect of analytical treatment interruption and reinitiation of antiretroviral therapy on HIV reservoirs and immunologic parameters in infected individuals
Source: PLoS Pathog. 2018 Jan 11;14(1):e1006792. doi: 10.1371/journal.ppat.1006792 (PMC5764487; doi:10.1371/journal.ppat.1006792)
Supplement: S3 Table — (PDF) [file ppat.1006792.s007.pdf]

S3 Table.

Genes associated with HIV life cycle and pathogenesis (2)			Interferon regulated genes (2)	Regulation of immune activation (3)
ABCE1	FOS	NUP85	ADAR	ATF3
ABI2	GML	PAK2	APOBEC3F	BCL3
ADAM10	GOLPH3	PDCD6IP	APOBEC3G	BCL6
AGFG1	HGS	PDIA6	APOBEC3H	C5orf13
AKT1	HTATSF1	PIAS4	BST2	CD70
APOBEC3F	IDH1	PML	DDX58	CDCP1
APOBEC3G	IFNAR1	PPIA	EIF2AK2	CDKN1A
APOBEC3H	IFNG	PRF1	IFI35	CYP2F1
ATM	IFNGR1	PSIP1	IFI6	DUSP10
ATR	IL10	PTPRC	IFIH1	DUSP4
BST2	IL10RA	RANBP1	IFIT1	DUSP8
BTRC	IL12B	RANBP2	IFIT3	EFCAB5
CCL11	IL18	RELA	IFITM1	GABARAPL1
CCL18	IL1A	RGP1	IFNAR1	GPR15
CCL2	IL1B	RICS	IFNAR2	IDI1
CCL4	IL1RN	SLC2A1	IFNG	IGFBP3
CCL5	IL2RA	SMARCB1	IFNGR1	IGFBP4
CCL7	IL4	SPTAN1	IFNGR2	LONRF1
CCNT1	IL4R	STAU1	IRF1	NAMPT
CCR2	IL8RA	STAU2	IRF9	PRRG2
CCR5	INPP5J	SUV420H1	ISG15	PSPH
CD209	IRF1	TCEB1	JAK1	SELK
CD28	IRF7	TCEB3	JAK2	SERTAD3
CD4	JAK1	THOC2	MX1	SLC2A14
CDC25C	KHDRBS1	TLR7	OAS1	SMAD7
CDK9	KLHDC2	TLR8	OAS2	TMEM166
CLEC4M	KPNB1	TLR9	OAS3	TNFSF9
COPB2	LCK	TNFRSF1A	OASL	TRAF4
CRAT	LCP2	TNPO3	PIAS1	
CTDP1	LIG4	TOMM70A	PSMB8	
CTLA4	LPL	TRAPPC1	PTPN2	
CUL5	LTA	TRIM22	RNASEL	
CX3CR1	LYPD4	TRIM32	SOCS1	
CXCL12	M6PRBP1	TRIM5	STAT1	
CXCR4	MAP3K5	TRIM55	STAT2	
CXCR6	MAP4	TSG101	TAP1	
DDX3X	MBL2	UBE2I	TRIM22	
DDX53	MED28	UNG	TRIM5	
DEFB1	MED4	VDR	TYK2	
DHX9	MED7	VPRBP	ZBP1	
DMXL1	MID1IP1	VPS4A		
DNM2	Med6	XPO1		
DYSF	NCOR2	XRCC5		
ETF1	NEDD4L	ZNF436		
FBXW11	NMT1	ZNF536		
FLNB	NUP153	ZNRD1		
