# Supplementary material for: Effect of analytical treatment interruption and reinitiation of antiretroviral therapy on HIV reservoirs and immunologic parameters in infected individuals
Source: PLoS Pathog. 2018 Jan 11;14(1):e1006792. doi: 10.1371/journal.ppat.1006792 (PMC5764487; doi:10.1371/journal.ppat.1006792)
Supplement: S4 Table — A two-tailed Wilcoxon matched-pairs signed rank test was performed with P values < 0.05 considered significant. (PDF) [file ppat.1006792.s008.pdf]

**S4 Table.**

| Cytokines/ chemokines | P value | Inflammation markers | P value |
|---|---|---|---|
| IL-1β | 0.9219 | APRIL/TNFSF13 | 0.4922 |
| IL-1Ra | 0.6953 | BAFF/TNFSF13B | 0.1934 |
| IL-4 | 0.7695 | sCD30/TNFRSF8 | 0.1602 |
| IL-5 | 0.4316 | sCD163 | 0.084 |
| IL-6 | 0.9219 | Chitinasae 3-like 1 | 0.1309 |
| IL-7 | 0.625 | gp130/sIL-6Rβ | 0.4922 |
| IL-8 | 0.7695 | IFN-α2 | 0.6406 |
| IL-9 | 0.9219 | IFN-β | 0.1934 |
| IL-10 | 0.4922 | IFN-γ | >0.9999 |
| IL-12 (p70) | 0.7871 | sIL-6Rα | 0.1309 |
| IL-13 | 0.5078 | IL-8 | 0.625 |
| IL-17 | 0.5566 | IL-11 | 0.4922 |
| Eotaxin | 0.7695 | IL-12 (p40) | 0.7422 |
| FGF basic | 0.4922 | IL-12 (p70) | 0.8262 |
| G-CSF | 0.6953 | IL-19 | >0.9999 |
| GM-CSF | >0.9999 | IL-28A/IFN-λ2 | 0.9219 |
| IFN-g | 0.9219 | IL-29/IFN-λ1 | 0.9219 |
| IP-10 | 0.1055 | IL-35 | 0.4922 |
| MCP-1 | 0.8652 | MMP-1 | 0.6953 |
| MIP-1α | 0.5566 | MMP-2 | 0.375 |
| PDGF-bb | 0.2754 | MMP-3 | 0.4922 |
| MIP-1β | 0.1602 | Osteocalcin | 0.4922 |
| RANTES | 0.0195 | Osteopontin | 0.3223 |
| TNF-α | 0.4922 | sTNF-R1 | 0.6523 |
| VEGF | 0.7695 | sTNF-R2 | 0.2754 |
| | | TSLP | 0.7695 |
| | | TWEAK/TNFSF12 | 0.6953 |
